# Plasmacytoma of the Cervix Possibly Caused by Neglected Pessary: A Case Report and Literature Review

**DOI:** 10.30699/ijp.2025.2061514.3465

**Published:** 2025-11-11

**Authors:** Noviana Nugrohowati, Bening Rahimi Titisari, Hanggoro Tri Rinonce, Rita Cempaka, Irianiwati Widodo Widodo, Tania Kusuma

**Affiliations:** 1 Department of Anatomical Pathology, Faculty of Medicine, Public Health, and Nursing, Universitas Gadjah Mada, Yogyakarta, Indonesia; 2 Academic Hospital Universitas Gadjah Mada, Yogyakarta, Indonesia; 3 Dr. Sardjito General Hospital, Yogyakarta, Indonesia

**Keywords:** Plasma Cell Neoplasm, Cervix Uteri, Pessaries, Pathology

## Abstract

**Background & Objective:**

Plasmacytoma is a rare plasma cell dyscrasia marked by localized monoclonal plasma cell proliferation, distinct from multiple myeloma by its lack of systemic symptoms. Extramedullary plasmacytoma (EMP), the rarest form, typically occurs in the upper respiratory tract. Cervical involvement is rare. Chronic inflammation may play a role in the development of cervical plasmacytoma, though this remains unproven for cervical cases.

**Case Presentation:**

We present a unique case of a 74-year-old woman who presented with vaginal bleeding linked to a neglected pessary inserted 15 years earlier. Upon examination, we discovered overgrown tissue with active bleeding. The biopsy revealed a monomorphic plasma cell infiltration, and immunohistochemistry confirmed CD138-positive, BCL2-negative cervical plasmacytoma, a rare occurrence in the cervix.

**Conclusion:**

This case highlights the importance of considering cervical EMP in atypical cytology and suggests a possible link to chronic pessary-induced inflammation. Early diagnosis through histopathology and immunohistochemistry is essential.

## Introduction

A plasmacytoma is a rare form of plasma cell dyscrasia, characterized by a localized proliferation of monoclonal plasma cells. It is distinguished from multiple myeloma, a more common dyscrasia, through the absence of a systemic disease, such as hypercalcemia, bone lesions, and renal failure (1). Plasmacytoma can be categorized into two groups based on location: solitary bone plasmacytoma (SBP), when arising in the bone, and extramedullary plasmacytoma (EMP), when occurring in soft tissue (1–3). While up to 80% of EMPs are located in the upper respiratory and digestive tracts, cases have also been reported in the urogenital tract, central nervous system, and female reproductive system (1,3–5). Several cases of plasmacytoma in Indonesia have been reported in the literature, however none involved the female reproductive tract (6–12). The risk factors of plasmacytoma are still unknown. Chronic inflammation is believed to be associated with plasma cell dyscrasias, and a previous report has examined its role in nasal EMP (13,14). However, no studies to date have investigated its role in the female reproductive tract, particularly the cervix.

We report this case to highlight its diagnostic challenges, as its clinical and pathological features can mimic those of other common gynecologic malignancies. We aim to raise awareness among clinicians and pathologists, encouraging consideration of plasmacytoma in the differential diagnosis of cervical lesions. Additionally, this report also explores potential risk factors, particularly chronic inflammation, to help identify patterns that support earlier recognition and more accurate diagnosis in future cases. This report is possibly the first documented case suggesting a neglected vaginal pessary as a potential chronic inflammatory risk factor for cervical plasmacytoma.

## Case Presentation

A 74-year-old woman was referred to our hospital with vaginal bleeding, suspected to be associated with a neglected pessary inserted 15 years prior. The bleeding began five days earlier, soaking two sanitary pads within the first two hours and accompanied by blood clots. She denied abdominal pain or abnormal vaginal discharge. Obstetric history noted menarche at 13, menopause at 54, three term pregnancies (two live births, and one intrauterine fetal death). She had used a routine 3-month injectable contraception.

**Fig. 1:**
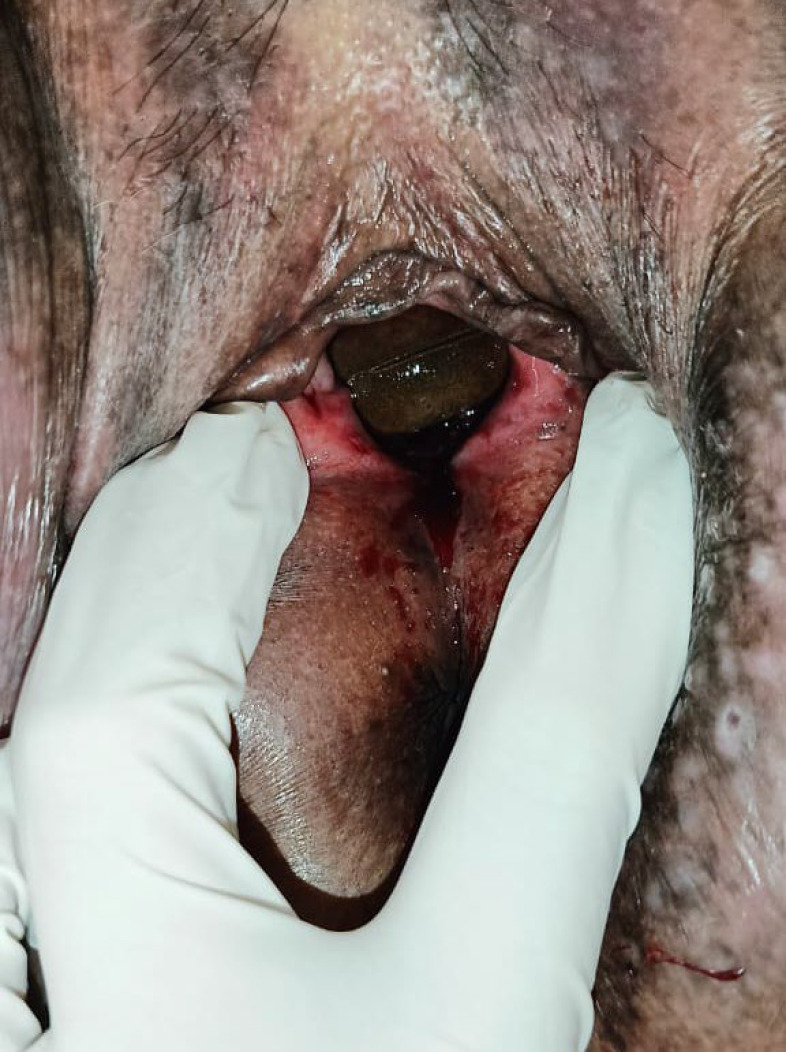
Clinical Presentation of Uterine Cervix Showing Active Bleeding

On examination, her blood pressure was mildly elevated at 154/85 mmHg. Abdominal findings were unremarkable. Speculum and bimanual examination revealed vulvar excoriation and a pessary within the vaginal canal, encased by overgrown tissue at the 10–11 o’clock position, with active bleeding and yellow-green discharge as seen in Figure 1.

Laboratory results showed mild anemia with a hemoglobin level of 11.1 g/dL, thrombocytosis of 496000/μl, elevated procalcitonin (0.43%), and hypoalbuminemia (3.93 g/dL), with normal renal function. Chest radiography and gynecologic ultrasound revealed no significant abnormalities.

**Fig. 2:**
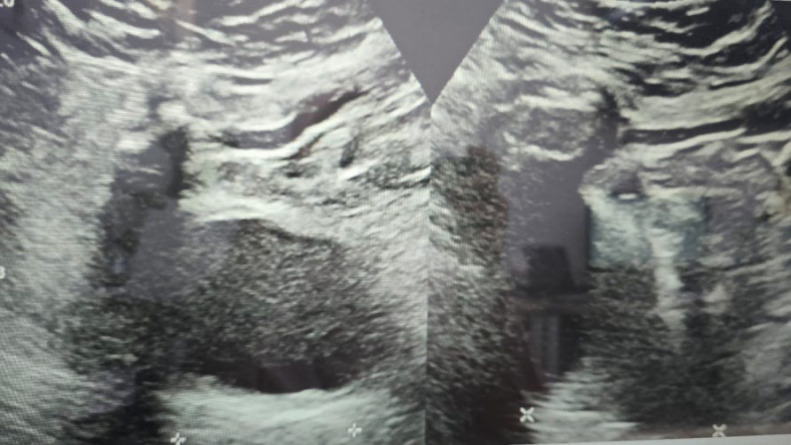
Ultrasound Examination. The uterus measured 3.89 × 4.86 cm, with no masses observed in either adnexa.

**Figure 3:**
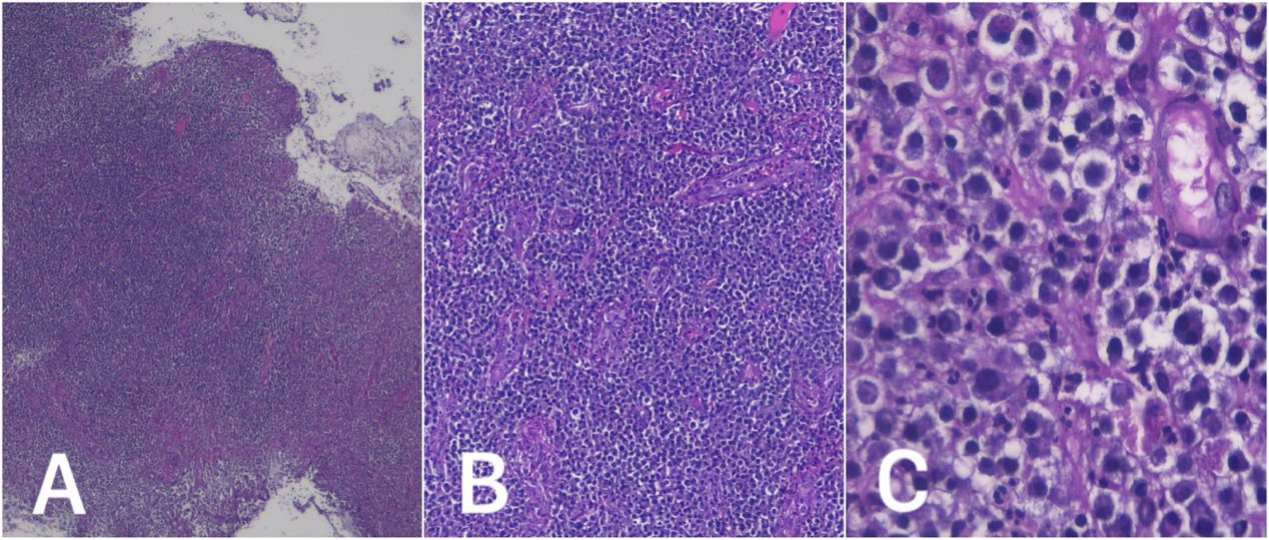
Hematoxylin & Eosin (H&E, 4x, 10x, 40x magnification) stained results. Histopathological examination revealed infiltration of monomorphic plasma cells.

**Figure 4:**
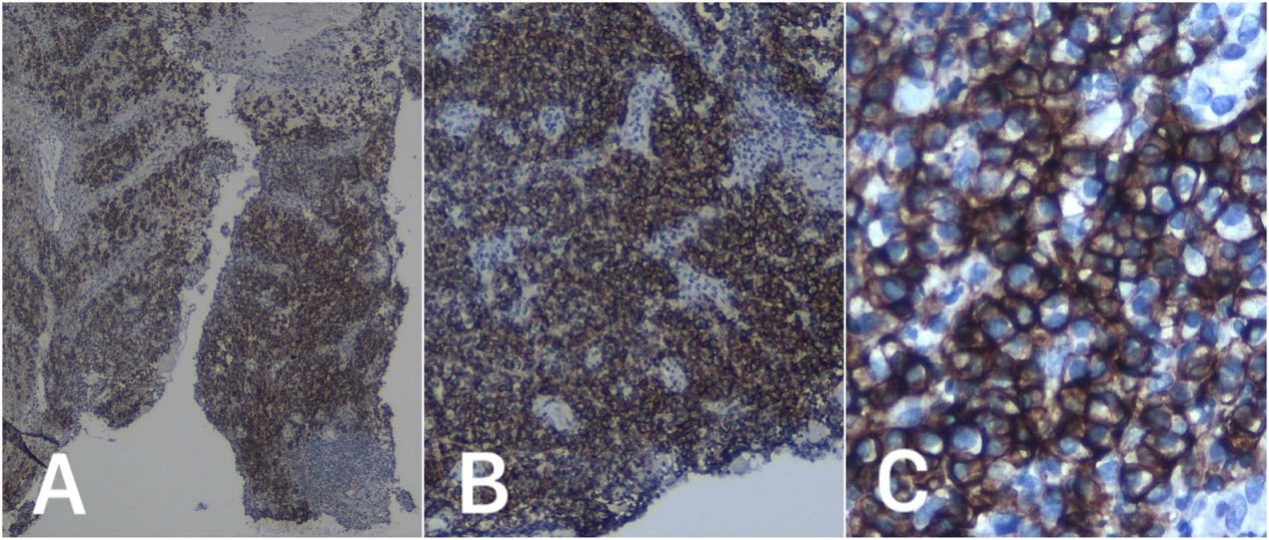
Immunohistochemistry results showed positive CD138 staining in the majority of tumor cells (4x, 10x, 40x magnification)

**Figure 5:**
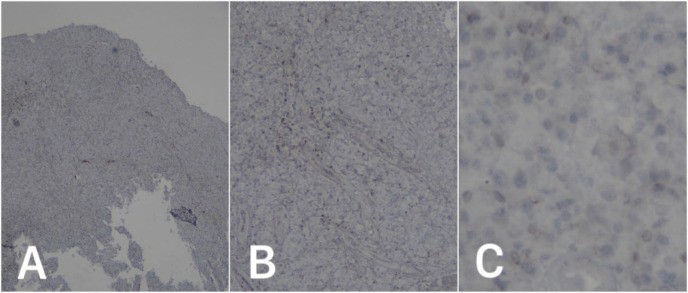
Immunohistochemistry results revealed negative staining for BCL2 (4x, 10x, 40x magnification).

Histopathological examination (Figure 3) of a cervical portio biopsy showed infiltration of monomorphic plasma cells, small to medium in size with moderate cytoplasm, round to oval eccentric nuclei, fine chromatin, with some hyperchromasia. Vascular proliferation with plump endothelial cells, vascular dilatation, erythrocyte extravasation, and a mixed infiltrate of lymphocytes and neutrophils were also observed. An immunohistochemical panel of CD138 and BCL2 was done, which demonstrated intense CD138 membranous staining in the majority of tumor cells and BCL2 negativity, confirming the final diagnosis of cervical plasmacytoma and ruling out differential diagnoses such as B-cell lymphoma and leukemia (Figures 4 and 5).

**Table 1:** Summary of Previous Plasmacytoma of the Cervix including Gynecological Risk Factors

Age	Clinical Manifestation	Possible Gynecologic Risk Factors	Pathology	IHC	Treatment	Reference
46	Cervical polyp, abnormal Pap smear	Family history of breast cancer	Atypical plasma cells	CD138 (+) Lambda light chain (+)κ-light chain (+)	Hysterectomy	(1)
21	Asymptomatic, abnormal Pap smear	None	Atypical plasma cells	CD138 (+) MUM-1 (+) Lambda light chain (+)	Radiotherapy	(15)
77	7 day vaginal bleeding	None	Well-differentiated plasma cells	CD138(+) CD38(+) κ-light chain (+)	Hysterectomy with postoperative radiotherapy	(16)
34	Postcoital bleeding, abnormal Pap smear	Intermittent oral contraceptivepills for 9 years	Atypical lymphoid cell proliferation	CD138 (+)MUM1 (+) CD19 (+)κ-light chain (+)	Total Abdominal Hysterectomy	(17)
38	Cervical polyp	None	Atypical plasma cells,plasmablast differentiation, tumor giant cells, atypical mitoticfigures.	CD138 (+) CD38 (+) λ-light chain (+)	Vaginal Hysterectomy	(18)
37	1 month irregular vaginal bleeding, endocervical polyp	Nulligravid	Atypical plasma cells, mitoses and multinucleated cells	λ-light chain (+)	Hysterectomy	(19)
45	Asymptomatic, abnormal Pap smear	History of uterine myoma	Atypical plasma cells with pleomorphic nuclei, bizarre giant cells	CD138 (+)λ-light chain (+) EMA (+) Vimentin (+)	Radiotherapy	(20)
74	5 day vaginal bleeding	History of IUFD and neglected pessary	Infiltration of monomorphic plasma cells	CD138 (+) BCL2 (-)	N/A	Present Case

## Discussion

Within the female reproductive tract, EMPs have been reported to be found in the ovary, cervix, and vagina (3). Limited to English literature published in the last 25 years, only seven cases of plasmacytoma of the cervix have been reported, as seen in Table 1.

EMP is an abnormal immunoproliferative neoplastic disease of monoclonal mature B-cell lineage, originating from a transformed plasma cell, and occurring outside the bone marrow (21). It is hypothesized that chronic irritation and inflammation may contribute to its pathogenesis, given the established role of inflammation in tumorigenesis (22). For instance, in cervical cancer, elevated levels of inflammatory cytokines such as IL-1β, IL-6, and IL-8 in the vaginal environment have been associated with the presence of malignancy or precancerous lesions (23,24). Additionally, previous literature on nasal EMP has suggested that recurrent inflammation may drive the rapid proliferation of B-lymphocytes, thereby increasing the risk of genetic mutations in these hematopoietic cells and potentially leading to tumor development (14). In the present case, the neglected pessary likely induced persistent mechanical trauma, local ischemia, and chronic infection, resulting in a pro-inflammatory microenvironment that may have facilitated neoplastic transformation. Moreover, previously reported cases of neglected vaginal pessaries have been associated with the development of cervical intraepithelial neoplasia (CIN), endometrial cancer, and vaginal cancer (25,26). However, to date, no reports have linked neglected pessaries with plasmacytoma of the cervix, likely due to the rarity of this neoplasm. This report suggests that a similar inflammatory mechanism may underlie the development of cervical EMP in this case.

Although previous reports of cervical EMP have not identified inflammation-related risk factors, several have noted gynecological factors typically associated with an increased risk of reproductive cancers. These include a family history of breast cancer, nulligravidity, and prolonged use of hormonal contraceptives (1,17,19). In our case, the patient had a history of regular 3-month injectable contraception containing depot medroxyprogesterone acetate (DMPA). While long-term DMPA use has been linked to a slight increase in breast cancer risk, its association with cancer in the cervix remains inconclusive (27).

The clinical presentation of cervical EMP is varied and often lacks specificity, which contributes to substantial diagnostic difficulties for clinicians. Patients may present with a wide spectrum of symptoms, the most common being abnormal vaginal bleeding as seen in our patient. This bleeding can manifest in several forms, such as intermenstrual bleeding, heavy menstrual bleeding, or more specifically, postcoital bleeding. In some cases, a cervical polyp may be observed upon pelvic examination, further complicating the differential diagnosis due to its similarity in appearance to other benign or malignant cervical lesions. Notably, a significant proportion of patients remain asymptomatic, and in these instances, cervical EMP is frequently detected incidentally. Routine cervical cancer screening procedures, especially Pap smear cytology, play a crucial role in such incidental findings, which Schor *et al.* have reported to have shown prominent inflammatory changes, atypical cells, and a possible low-grade intraepithelial lesion, which may initially raise concerns for other premalignant or malignant conditions and lead to misdiagnosis of cervical cancer as seen in the report by Wang *et al. *(15,16,20). 

Histopathological examination of plasmacytoma typically reveals dense infiltration of plasma cells displaying variable degrees of maturation and atypia. In previously reported cases, histology has frequently shown atypical plasma cell infiltration, sometimes accompanied by atypical giant cells and abnormal mitotic figures, although not observed in our case. However, histology alone is insufficient to confirm the diagnosis, as it cannot reliably distinguish between reactive plasma cell infiltrates and true neoplastic monoclonal proliferation. To confirm the diagnosis, immunohistochemistry is essential. In most cases, tumor cells show strong positivity for plasma cell markers such as CD138 and CD38, resembling the immunophenotypic profile seen in multiple myeloma. Immunohistochemistry or in situ hybridization can also be used to identify kappa- or lambda-light chains restrictions to confirm monoclonality of the plasma cells. However this was not done in our case due to the unavailability of resources in our hospital. Additionally, the diagnosis of EMP is supported by the absence of serum and urine M-protein, less than 5% of bone marrow involvement, normal skeletal survey, and no end-organ damage to rule out multiple myeloma. 

Given the overlap in clinical and cytological features, cervical EMP is commonly misinterpreted as more prevalent gynecologic conditions such as chronic cervicitis and cervical intraepithelial neoplasia (CIN) (16). In our case, the patient initially presented with cytologic findings consistent with chronic inflammation and was suspected of having plasma cell cervicitis, a benign condition with overlapping histologic features. The definitive diagnosis of cervical EMP was ultimately established through immunohistochemical staining, which revealed monoclonal plasma cell proliferation positive for CD138. This highlights the critical role of immunohistochemistry in distinguishing EMP from benign plasma cell infiltrates or other cervical lesions, especially when histopathology alone is inconclusive. Previously reported cases have also shown positivity for MUM-1, EMA, Vimentin, CD19, and CD38.

As EMP is a highly radiosensitive malignancy, most authors agree that radiotherapy is the treatment of choice, especially in regions inaccessible by surgery, such as the head and neck. However, due to its rarity, no standardized treatment protocol has been established. In practice, a typical radiotherapy regimen consists of 40–50 Gy administered over approximately four weeks (16). When the EMP is located in easily accessible areas such as the digestive and reproductive tracts, surgery can also be considered. Among the previously reported cases, four cases were treated with hysterectomy alone, two with radiotherapy, and one with hysterectomy followed by postoperative radiotherapy. In terms of chemotherapy, previous studies have shown no benefit. 

The prognosis for EMP is generally favorable, particularly when compared to SBP, and is significantly improved with early diagnosis and treatment. Around 30% of EMP cases progress to multiple myeloma within 10 years, with a 5-year survival rate of 100%. In contrast, nearly 50% of SBP cases progress to multiple myeloma, and the 5-year survival rate drops to just 33% (1). From all previous reported cases, none have reported progression into multiple myeloma.

A key limitation of this case is the absence of confirmatory investigations such as serum protein electrophoresis, bone marrow aspirate, and free light chain analysis, which are essential for excluding systemic involvement and establishing a definitive diagnosis. The unavailability of these diagnostic modalities limited our ability to rule out multiple myeloma or other systemic plasma cell dyscrasias, making the diagnosis predominantly reliant on histopathology and immunohistochemistry.

## Conclusion

Given the limited number of reported cervical EMP cases in the literature, our case highlights the importance of considering cervical EMP in atypical cytology. Early and accurate diagnosis through histopathology and immunohistochemistry is essential for appropriate management and improved prognosis. Additionally, this case highlights a potential association between neglected pessary-induced inflammation and the development of cervical carcinoma, particularly plasmacytoma.
